# Study on the Performance of Epoxy-Modified Asphalt and Steel Slag Ultra-Thin Friction Course

**DOI:** 10.3390/ma17184513

**Published:** 2024-09-13

**Authors:** Quanmin Zhang, Ziyu Lu, Anqi Chen, Shaopeng Wu, Jianlin Feng, Haiqin Xu, Yuanyuan Li

**Affiliations:** 1 State Key Laboratory of Silicate Materials for Architectures, Wuhan University of Technology, Wuhan 430070, China; quanmin@whut.edu.cn (Q.Z.); fengjianlin@whut.edu.cn (J.F.); xuhaiqin@whut.edu.cn (H.X.); 2 School of Transportation and Logistics Engineering, Wuhan University of Technology, Wuhan 430063, China; 3 School of Civil Engineering and Architecture, Wuhan Institute of Technology, Wuhan 430074, China; liyy@wit.edu.cn

**Keywords:** ultra-thin overlay, road maintenance, epoxy resin, steel slag, skid resistance

## Abstract

Ultra-thin overlays (UTOL) are a standard highway pre-maintenance method used to improve the road surface performance of asphalt pavements and to repair minor rutting and cracking. However, the thin thickness makes it very sensitive to external changes, which increases its wear and shortens its life. So, this paper aims to prepare a durable and skid-resistance asphalt ultra-thin overlay using epoxy asphalt (EA) and steel slag. First, the physical properties of EA were characterized by penetration, softening point, flexibility, and kinematic viscosity tests. The dynamic shear rheometer (DSR) test characterizes EA’s rheological properties. Differential Scanning Calorimetry (DSC), kinematic viscosity, and Fourier transform infrared spectroscopy (FTIR) characterized the EA’s curing process. Finally, the pavement performance of an epoxy ultra-thin overlay (EUTOL) prepared with EA and steel slag was tested. The results show that the epoxy resin particles increase with the increase in epoxy resin dosage, and at 40%, its epoxy particles are uniformly distributed with the most significant area share. With the addition of epoxy resin, the needle penetration of EA decreases and then increases, the flexibility decreases at a slower rate, and the softening point rises significantly. Moreover, the growth of the elastic component in EA significantly improved the high-temperature viscoelastic properties. Considering its physical and rheological properties, the optimal doping amount of 40% was selected. By analyzing the curing behavior of EA (optimum dosage), the combination temperature of EA is 150 °C, which meets the needs of mixing and paving asphalt mixtures. After 12 h of maintenance at 120 °C, its reaction is sufficient. The skid-resistance durability, high-temperature, low-temperature, water stability, and fatigue resistance of UTOL can be effectively improved using steel slag coarse aggregate.

## 1. Introduction

Asphalt pavement has good continuity, driving comfort, stable seismicity, and easy maintenance with low noise, and is widely used in municipal pavements, motorways, airport runways, and other scenes [1,2,3,4]. By the end of 2023, the total mileage of China’s highways will reach 5,436,800 km, and the mileage of road maintenance will reach 5,431,300 km, accounting for 99.9% of the road mileage [5]. However, with the significant growth of traffic volume and the development trend of heavy traffic load, asphalt pavement often produces rutting, transverse cracks, longitudinal cracks, and other diseases, affecting driving safety and reducing the pavement service life.

Preventive maintenance techniques are proven high-performance and effective, with high economic and environmental benefits [6,7]. Usually, it is used to improve the pavement’s overall performance (including anti-skid, anti-rutting, and anti-cracking properties) [8,9,10]. Pavement pre-maintenance practices generally include fog seals [11], slurry seals [12], gravel seals [13], micro-surfacing [14], and ultra-thin overlays [15]. UTOL conservation technology is more comprehensive [6]. As an overlay, UTOL is used to maintain old pavement surfaces and construct new roadways [16]. In addition, UTOL has significant functional characteristics: low energy consumption [17], low pollution [18], low traffic interference [19], high cost effectiveness [11], and so forth. So, it is one of the most commonly used maintenance techniques. However, UTOL is typically a temperature-sensitive material prone to rapid degradation when exposed to oxygen, ultraviolet radiation, and heavy traffic loads over long periods [20]. It degrades rapidly due to its ultra-thin structure and drastic temperature fluctuations [21,22]. Therefore, there is a need to develop a durable and wear-resistant ultrathin cover layer.

Epoxy resin and epoxy curing agent performed an irreversible curing reaction and formed a crosslinked structure filled in the asphalt binder [23,24,25]. Wu et al. found that the EA will undergo phase reversal from a fractured structure to a network crosslinked structure with an increasing dosage of epoxy resin [26]. EA has the characteristics of high tensile strength, temperature stability, corrosion resistance, and fatigue resistance compared to other modifications [27]. Wei Huang et al. studied the properties of EA mixtures under different influencing factors, and the results indicated that the addition of epoxy resin could significantly enhance the stability and splitting strength of the mixtures [28]. Sprinkle-on et al. applied EA to an ultra-thin overlay and conducted a durability evaluation study on the pavement after six years of use; the results showed that it not only had excellent anti-skid properties but also had a service life of up to 15 years and good water resistance [29].

Using natural aggregates, which make up more than 90% of asphalt mixes, is environmentally damaging and expensive [30]. In addition, steel manufacturing leads to the production of large volumes of metallurgical waste (steel slag). China produces over 100 million tons of steel slag annually, but the usage rate is less than 8% [31,32]. Steel slag has the potential to replace natural aggregates based on its good anti-abrasiveness, high strength, high alkalinity, and good adhesion to asphalt [33]. Murmu et al. found that steel slag has higher mechanical strength than natural aggregates and can form a strong backbone structure in asphalt mixtures, and that the use of steel slag aggregates improves the toughness of pavements [17]. Divadari et al. utilized scanning electron microscopy to evaluate the physical external morphology and the pore structure characteristics of steel slag [5]. The results showed that the steel slag surface had a plentiful texture, which enhanced the bond power between the asphalt and aggregate and improved the abrasion resistance. Pattanaik et al. researched the mechanical properties of surfaces using open-graded asphalt mixtures prepared by replacing a portion of the aggregate with steel slag. The study showed that rutting, cracking, and fatigue resistance were improved [34]. Many researchers have tested the abrasion resistance of steel slag, indicating that the steel slag’s abrasion resistance is about 15–30% better than that of natural aggregates [35]. Li et al. showed that preparing UTOL provides better abrasion resistance, skid resistance, and environmental protection using steel slag [36].

**Figure 1:**
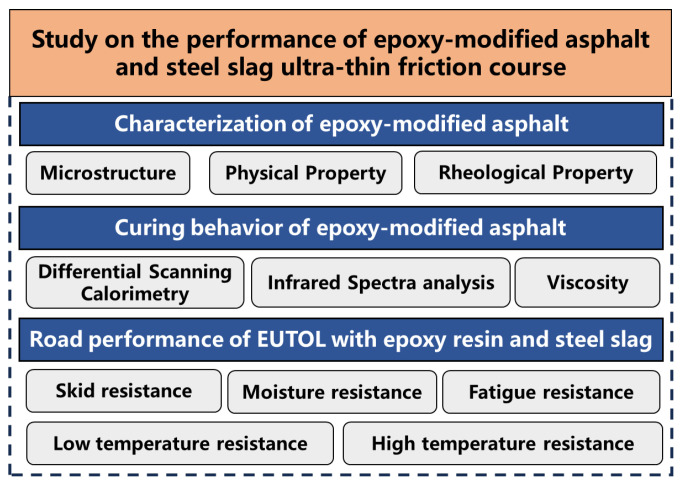
Technology roadmap of EUTOL.

In summary, using EA and steel slag can improve the durability and abrasiveness of ultra-thin overlays. Therefore, this paper explores the feasibility of preparing ultra-thin abrasive layers from steel slag and epoxy resin by investigating using steel slag as a replacement for natural aggregates and the preparation of EUTOL using AC-5 gradation. In order to overcome the above limitations, this paper focuses on the following three aspects: (1). By studying the microscopic and physical characteristics and rheological behavior of different dosages of EA to determine the appropriate dosage of epoxy resin. (2) The processes for EUTOL preparation were determined by studying the EA curing behavior. (3) Using steel slag instead of basalt to prepare EUTOL and investigate its performance. Figure 1 illustrates the design process.

## 2. Materials and Methods

### 2.1. Materials

#### 2.1.1. Asphalt

The asphalt used for this paper is 70# base asphalt, and its basic performance indexes are shown in Table 1.

#### 2.1.2. Epoxy Resin

**Figure 2:**
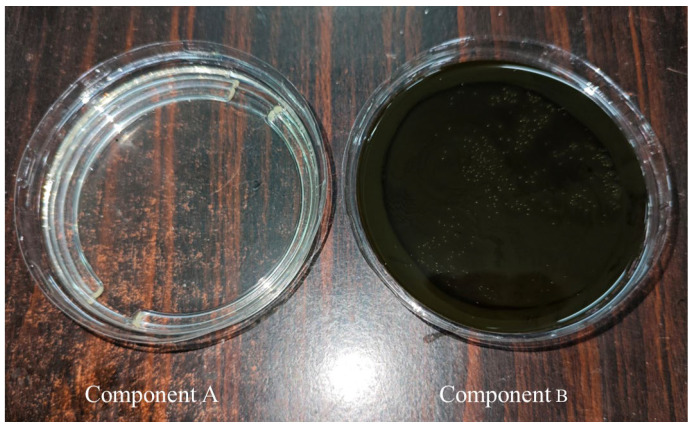
Epoxy resin A component and B component.

The epoxy resin used in this study consisted of a 1:3 mixture of epoxy resin component A (bisphenol A epoxy resin) and curing agent component B (curing agent, reclaimed asphalt light component, and its additives). The basic performance and appearance are shown in Table 2 and Figure 2.

#### 2.1.3. Aggregate Sand Fillers

Basalt was selected as the natural aggregate, and steel slag from a steel mill in Wuhan was used as the substitute aggregate. The aggregate was divided into fine aggregate (0–2.36 mm) and coarse aggregate (2.36–4.75 mm). The technical specifications of steel slag and basalt aggregates with different particle sizes are shown in Table 3. The filler is limestone mineral powder.

### 2.2. Sample Preparation

#### 2.2.1. Preparation of Epoxy-Modified Bitumen

**Figure 3:**
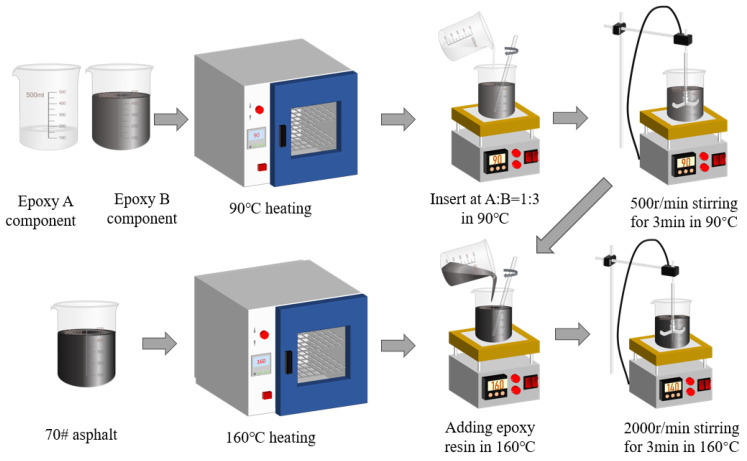
Flow chart of EA preparation.

EA is made by mixing epoxy resin and asphalt evenly in a particular proportion. The preparation process was as follows: (1) In an oven at 90 °C, heat the epoxy resin components A and B to soften them. (2) combine the epoxy A and B components in a beaker at a 1:3 mass ratio. (3) Stir the epoxy resin with a stirrer (500 rpm) for 3 min at 90 °C. (4) Heat the bitumen at 160 °C. (5) Pour the epoxy resin in the 10%, 20%, 30%, 40%, and 50% ratio into the bitumen beaker at 160 °C. (6) Stir the EA with a stirrer (2000 rpm) at 160 °C. The flow chart of epoxy-modified asphalt preparation is shown in Figure 3.

#### 2.2.2. Preparation of the Asphalt Mixtures

**Figure 4:**
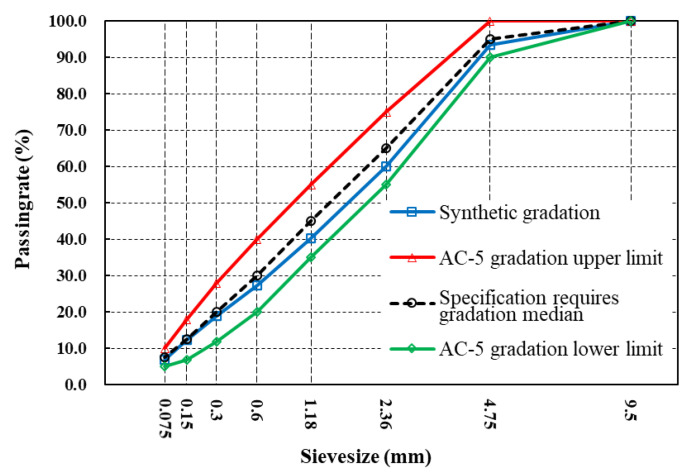
Gradation diagram of the AC-5 asphalt mixture.

Compared to other types of thin-covering, the OUTL preventive maintenance technique has a specific economic cost advantage in that the ultra-thin thickness of the thin-covering technique is generally a maximum of 25 mm with a low aggregate dosage [6]. In order to ensure the structural strength and skid resistance of asphalt mixtures, AC-5 gradation was used to design the asphalt mixtures in this study. Figure 4 illustrates the grade curves. The oil–rock ratio was considered 6.2%, and the whole basalt EUTOL-BB was prepared using limestone mineral powder as filler. Subsequently, EUTOL-BS was prepared by replacing coarse aggregate basalt with steel slag coarse aggregate by the iso-volumetric method; EUTOL-SB was prepared by replacing fine aggregate basalt with steel slag fine aggregate by the iso-volumetric method; and EUTOL-SS is prepared using the iso-volumetric method with steel slag aggregate replacing all basalt aggregate. The effects of different particle sizes of steel slag on pavement performance were investigated by preparing Marshall specimens, high-temperature rutting specimens, beamlet bending specimens, and semicircular fatigue specimens and conducting relevant asphalt mixture experiments. In order to investigate the effect of replacing natural aggregate particle size with steel slag aggregate on pavement performance, Marshall specimens, rutting specimens, trabecular bending specimens, and semicircular fatigue specimens were prepared in this study and characterized by relevant asphalt mixture experiments.

### 2.3. Test Methods

#### 2.3.1. Fourier Transform Infrared Spectroscopy

A Frontier IR/FIR (Bruker Optics (China), Beijing, China) Fourier transform infrared spectrometer was used in this test. All samples were prepared using the potassium bromide smear method, and the quantitative samples were dissolved in carbon disulfide and then titrated on a KBr wafer using a burette. Afterward, the solvent was allowed to volatilize and air-dry, i.e., the device was tested in a fixed window of the instrument. EA’s samples were scanned 32 times with a resolution of 2 cm^−1^.

#### 2.3.2. Fluorescence Microscopy Test

In this paper, a BXF-150 falling-type fluorescence microscope (Shanghai Binyu Optical Instrument Co., Ltd., Shanghai, China) was used. The epoxy resin asphalt was cured at 120 °C for 24 h. A 10× eyepiece and a 40× objective were used. Observe the epoxy curing reaction process and its distribution state. Samples were prepared without coverslips to ensure the reproducibility and stability of the EA’s microstructure.

#### 2.3.3. Differential Scanning Calorimetry Test

DSC experiments were conducted using a Discovery DSC2500 Differential Calorimetric Scanner (TA Instruments Inc., New Castle, DE, USA) to measure the rate of heat absorption and heat removal of epoxy resins and their modified asphalt materials as a function of temperature. The behavior of the EA curing reaction was investigated by analyzing the changes in heat flow caused by the curing reaction inside the EA.

#### 2.3.4. Asphalt Testing

In this paper, the asphalt tests are shown in Table 4.

#### 2.3.5. Asphalt Mixture Performance Testing

In this paper, the asphalt mixture tests are shown in Table 5.

## 3. Results and Discussion

### 3.1. Mechanistic Analysis of Epoxy Resin and Modified Bitumen

#### 3.1.1. Epoxy Asphat Microstructure

**Figure 5:**
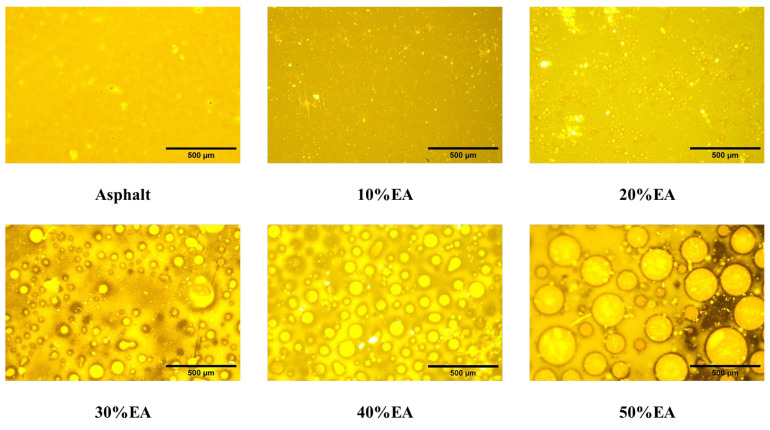
Fluorescence images of Asphalt and different EA.

The shape and the position of the epoxy particles of EA with different epoxy resin dosages were observed by fluorescence microscopy experiments. The fluorescence images are shown in Figure 5. The fluorescent phase in the figure is epoxy resin, and the background is matrix asphalt. As the dosage of EA increases, the epoxy resin particles gradually become larger and more uniformly distributed. Still, at 50% dosage, the epoxy resin particles become larger, and the distribution begins to disperse.

**Figure 6:**
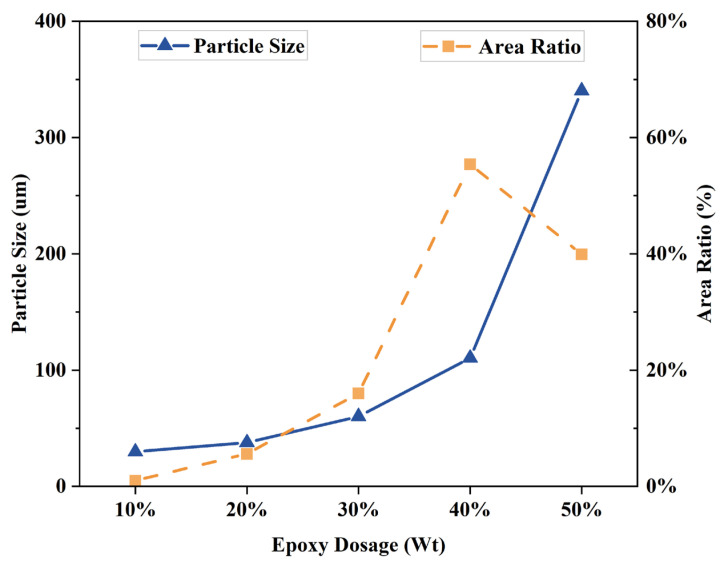
Particle size and area share of different dosages of EA.

The particle size of the EA particles was determined using an image analysis program (ImageJ 1.45) and statistically analyzed for epoxy particle size and area ratio. The results are shown in Figure 6. The epoxy resin dosage was increased from 10% to 40%, and the epoxy resin particles were increased from 30 microns to 140 microns. From 40% to 50%, the epoxy resin particle size increases sharply from 140 µm to 440 µm. In addition, the epoxy resin particle area increases from 3% to 60% when the epoxy resin is used from 10% to 40%. However, when the epoxy resin dosage reaches 50%, the epoxy resin particle area decreases to 42.9%. This difference is because the proportion of epoxy resin in the EA gradually increases, the epoxy resin undergoes an aggregation phenomenon during the reaction process, and the diameter of the particles increases sharply. However, the area ratio of the epoxy resin decreases. Therefore, the epoxy particles will be uniformly dispersed in the bitumen when the dosage of epoxy is 40%. During the curing reaction of epoxy resins, crosslinking and polymerization reactions occur between the unsaturated double bonds of the epoxy molecular and the double bonds of the curing agent. The linear long-chain molecules form chemical bonds and crosslink into a three-dimensional network structure [6,24]. With increased epoxy resin doping, the three-dimensional network structure gradually becomes larger.

#### 3.1.2. Physical Properties of EA

**Figure 7:**
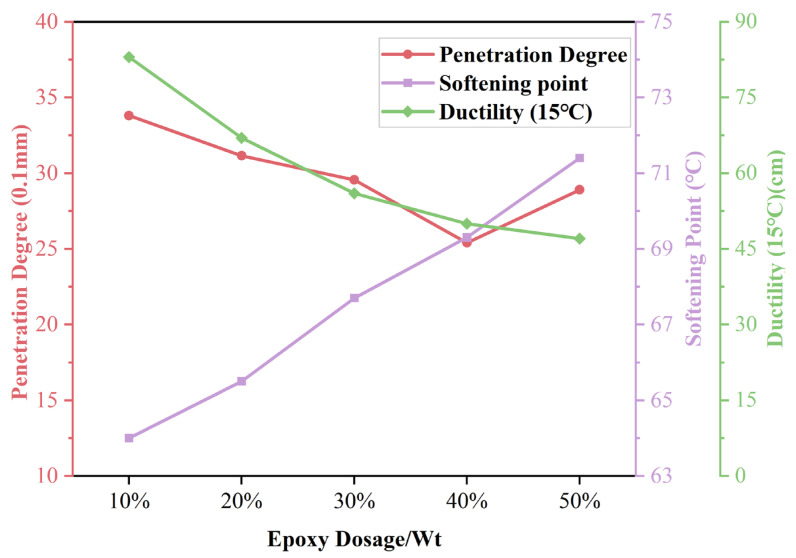
Physical properties of different dosages of EA.

In this study, to analyze the differences in the physical properties of different dosages of EA, consistency performance was analyzed by the penetration test, shifting performance was analyzed by the softening point test, and plasticizing performance was analyzed using ductility. The results are shown in Figure 7. The EA’s penetration decreased with the amount of epoxy resin from 10% to 40%. This shows that epoxy resin improves asphalt consistency and resistance to shear damage. However, the penetration degree increased when the epoxy resin dosage reached 50%. This is because the epoxy resin B component has a large amount of asphalt light component. The epoxy resin’s light component improves the EA’s penetration more than the epoxy crosslinking cure reduces the penetration. The EA’s softening point increases with the dosage of epoxy resin used. This is due to the thermosetting characteristics of the epoxy resin, which cures in the bitumen to form a stable crosslinked structure. Notably, the softening point of EA did not form a peak when the epoxy resin doping was 40%. This indicates that EA’s high-temperature stability depends only on the epoxy resin content, while the dosages of asphalt lightweight components do not affect EA’s temperature sensitivity. The ductility of the EA decreases with increasing dosage. The results show that EA’s ductility increases with the increase in epoxy resin dosage, and its resistance to plastic deformation decreases continuously. Therefore, it is because the epoxy resin is thermosetting, and the completion of the reaction will reduce the toughness of EA, which will reduce the low-temperature ductility of EA. It is easy to cause plastic fractures under the action of external forces. However, the increase in the dosage of epoxy leads to an increase in the content of the asphalt light component, which gradually decreases the decrease in the EA ductility. The asphalt light component may change EA’s spatial structure formation. Combining the above results, when the dosage of epoxy resin is 40%, EA’s penetration is the smallest, and consistency performance is the best; when the dosage is 50%, EA’s softening point is the largest, and shifting performance is the best. Since ductility is an inherent property of epoxy resins, EA’s ductility is weakened with increased epoxy resin dosage. However, the effect of the reduction diminishes with the increase in dosage. Thus, the best overall physical properties were obtained at 40% EA.

#### 3.1.3. Rheological Properties of EA

**Figure 8:**
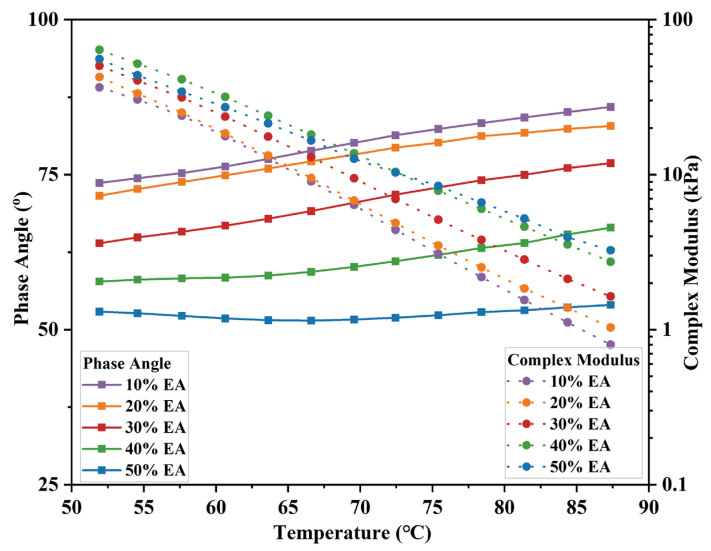
Phase angle and complex modulus of different dosages of EA.

In this study, EA’s mechanical properties were analyzed using a dynamic shear rheometer with temperature scans from 52 °C to 88 °C. The results are shown in Figure 8. EA’s phase angle (δ) grows, and the complex modulus (G*) declines with rising temperature. This is due to the transition of EA from a highly elastic state at low temperatures to a viscous flow state at high temperatures. EA’s maximum shear stress reduces, and its maximum shear strain grows, so G* decreases. Meanwhile, δ of EA is enhanced with rising temperature, which increases EA’s viscous component. Because the space structure formed by the epoxy resin increases the elastic component in the EA, resulting in a decrease in its δ. Moreover, EA’s G* gradually increases when the epoxy resin content is 10–40%. However, with 50% epoxy resin, the G* is lower than 40% EA at lower temperatures and gradually rises above 40% EA with increasing temperatures. As the dosage of epoxy resin increases, the EA content of the asphalt light component also increases, resulting in a decrease in G*. With increasing temperature, EA’s high-temperature deformation resistance has a progressively greater influence, and its G* decreases continuously.

**Figure 9:**
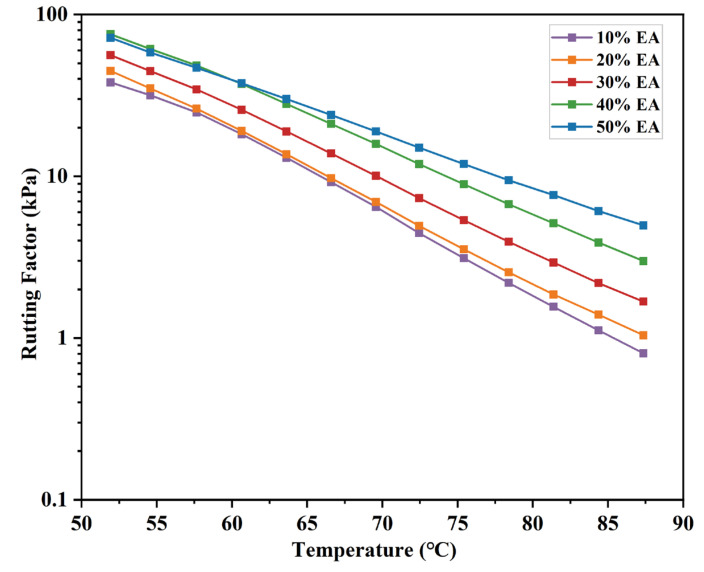
Rutting factor of different dosages of EA.

To better identify the effect of EA dosage on asphalt rheological characteristics, introduce the rutting coefficient (G*/sinδ) as EA’s evaluation index of anti-temperature rutting performance. The result is shown in Figure 9. G*/sinδ reduces with temperature elevation and is larger with EA dosage. It indicates that EA’s rutting resistance continues to decline with increasing temperature. However, 40% EA has a higher rutting coefficient than 50% EA at low temperatures. In conclusion, adding epoxy resin can substantially improve EA’s high-temperature rheological properties. Although 50% EA has the best high-temperature rutting resistance, 40% EA has better high-temperature deformation resistance and viscoelastic properties, which can be regarded as 40% EA having the best high-temperature rheological properties.

### 3.2. Study EA’s Curing Behavior

#### 3.2.1. Study EA’s Curing Temperature

**Figure 10:**
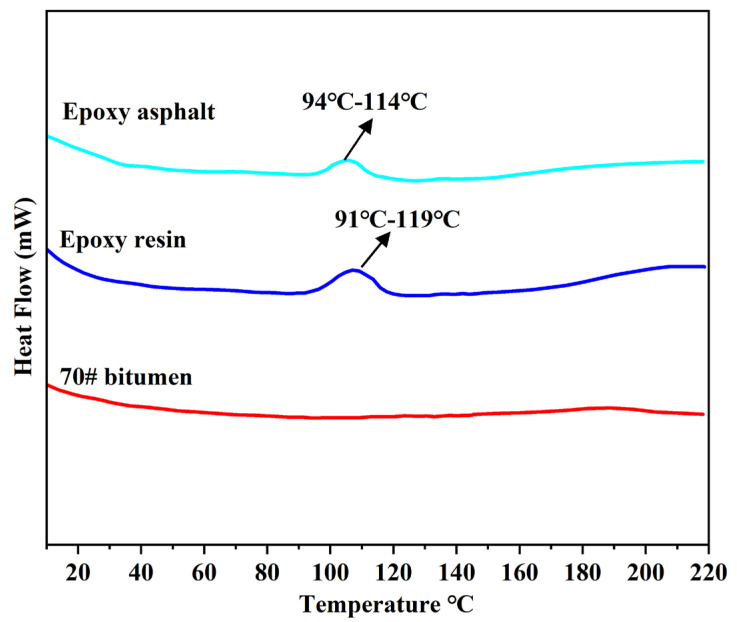
DSC curves of the matrix asphalt, epoxy resin, and epoxy bitumen.

In this study, the heat flow rate variation curves of 40% EA, epoxy resin, and matrix asphalt were determined by the DSC test, as shown in Figure 10. The relatively flat change in the heat flow rate of matrix asphalt indicates that the matrix asphalt does not react in the process. Meanwhile, the DSC curves of epoxy resin and EA have a reaction occurring at 90 °C–120 °C. At this time, the enthalpy of the epoxy resin and its modified bitumen increases and reaches its highest point when the temperature rises to 107 °C. The enthalpy of the epoxy resin and its modified bitumen increases when the temperature rises to 107 °C. It can be assumed that the presence of the bitumen does not affect the peak curing temperature of the epoxy resin. This indicates that EA is cured at temperatures ranging from 94 °C to 114 °C.

#### 3.2.2. Study EA’s Curing Process of Viscosity

**Figure 11:**
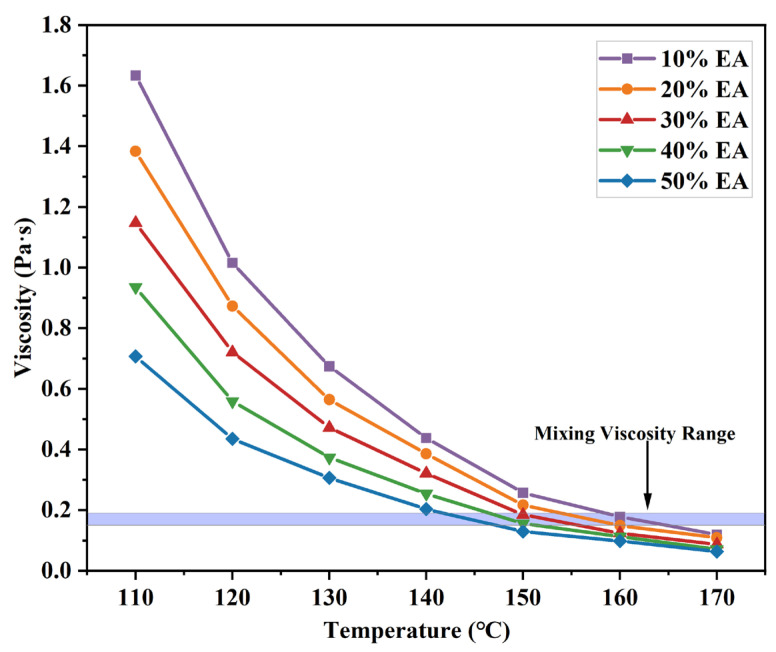
Viscosity versus temperature curves of different dosages of EA.

Viscosity is an important parameter in measuring asphalt as it is important in curing, machining, blending, compaction, and supplying. EA variation with temperature at different dosages is shown in Figure 11. EA viscosity decreases with an increase in addition because epoxy resin is less viscous than asphalt, and the addition of epoxy resin at the beginning of the reaction decreases EA viscosity. Generally, asphalt is mixed at a viscosity of 0.17 Pa∙s ± 0. 02 Pa∙s. The optimum EA dosage of 40% was determined above, and the temperature was 146–153 °C at 0.17 Pa∙s ± 0.02 Pa∙s, so 150 °C was chosen as the stirring temperature.

**Figure 12:**
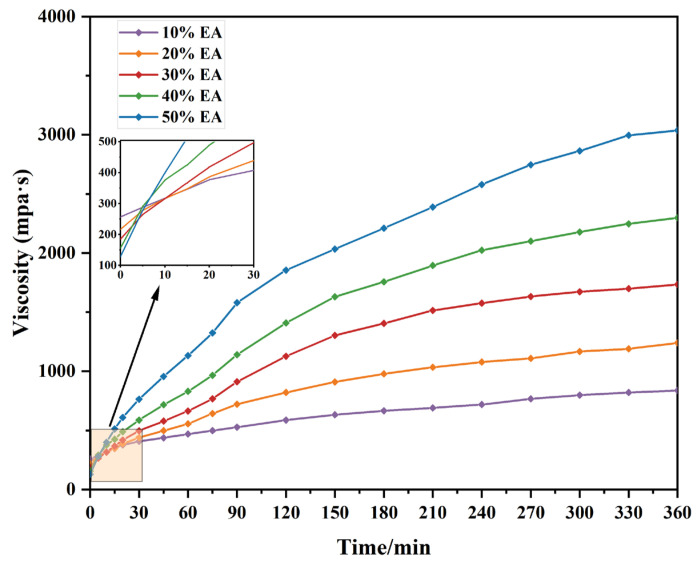
Time dependence curve of EA viscosity with different dosages.

However, unlike matrix asphalt, EA’s curing time is prolonged and influenced by mixture volume and temperature, among other factors. Moreover, the suitable viscosity of EA should be less than 3 Pa⋅s when compacting the mix. Therefore, studying the viscosity profiles of different EA with time at 150 °C made it possible to determine that EUTOL had enough time to be transported and paved. The results are shown in Figure 12. EA’s viscosity increases with the testing time and the proportion of epoxy resin dosages. Epoxy resin has two components in the curing reaction in the early stage of rapid reaction in the matrix asphalt to form a three-dimensional network structure. The asphalt viscosity within a short period of time increased rapidly; with the continuation of the curing reaction, the epoxy resin and curing agent content were reduced, the concentration was reduced, and the viscosity of the asphalt slowed down the growth rate [44]. The time taken to form microgels is shorter when the amount of epoxy resin increases. However, it can be obtained that at 150 °C, 40% EA has enough time for pavement construction treatment.

#### 3.2.3. Study EA’s Curing Process over Time

**Figure 13:**
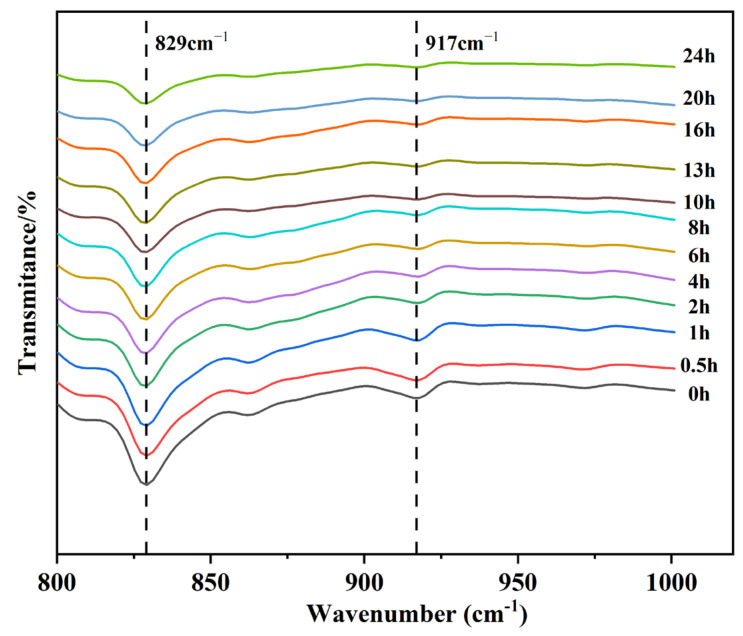
Local infrared images of 40% EA with different curing times.

**Figure 14:**
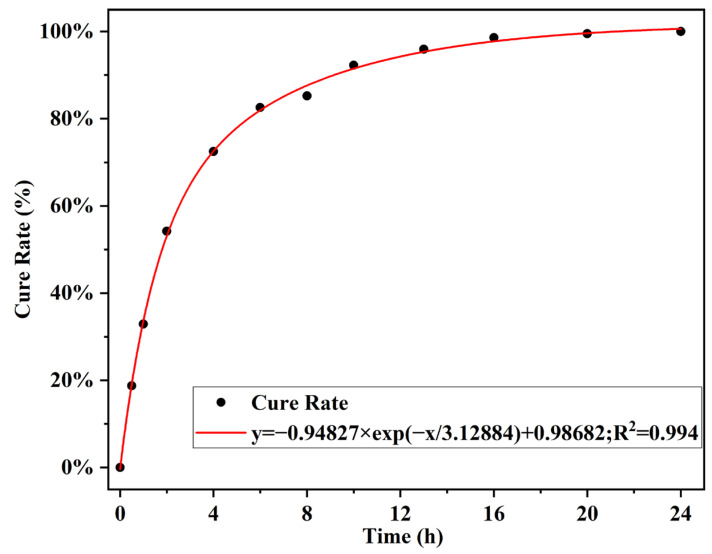
40% EA’s conversion rate changes over time.

In this paper, FILT is used to characterize the curing behavior of EA at 120 °C. The test results are shown in Figure 13. The epoxy group (917 cm^−1^) and benzene ring (829 cm^−1^) are characteristic peaks unique to epoxy resins and not found in asphalt. It was shown that the characteristic benzene ring peaks do not participate in the reaction during the curing process. The curing behavior of 40% EA was analyzed using Equations (1) and (2). The results of the calculations are shown in Figure 14.

(1) $$\alpha_{t}=1-\frac{A_{t}^{\prime }/A_{t}}{A_{0}^{\prime }/A_{0}}$$

(2) $$\beta_{t}=\frac{\alpha_{t}}{\alpha_{24}}$$

- $A_{t}^{\prime }$—The characteristic peak area of the epoxy group at time *t*;
- $A_{0}^{\prime }$—The characteristic peak area of the epoxy group at the beginning;
- $A_{t}$—Characteristic peak area of the benzene ring group at time *t*;
- $A_{0}$—Characteristic peak area of the benzene ring group at the beginning;
- $\alpha_{t}$—Epoxy group conversion at *t*,%;
- $\alpha_{24}$—Epoxy group conversion at 24 h,%;
- $\beta_{t}$—Curing degree of EA at *t*,%

The EA curing process is mainly in three stages: Accelerate the curing period for 0–4 h, the polymerization of hydroxyl and anhydride open rings within the epoxy molecule to generate a monoester, and the reaction of carboxyl and monoester within the epoxy to form a diester structure. In this stage, the EA reaction is rapid, and the conversion rate has reached 69%. The curing continuation period is 4–10 h, and the structure formed in the system due to the continuation of the reaction has not yet occurred—a large number of epoxy crosslinking, crosslinking system further diffusion. At this stage, the primary reaction of EA is completed, and the conversion rate has reached 95%. After 10 h for the curing and stabilization period, the epoxy reaction is complete, and the crosslinked system forms epoxy microcolloids. The mix mixing, transportation, and rolling paving processes should occur during the early stages of the curing reaction when the reticulation has not yet been formed, with high reactivity and fast strength growth. The high reactivity and the rolling process will not negatively affect the final strength. Therefore, the mix has enough construction time, and the mix with 12 h curing time has high enough strength.

### 3.3. EUTOL Pavement Performance

In summary, the optimum dosage of EA is 40% at a mixing temperature of 150 °C. Moreover, the working time at 150 °C is sufficient. Therefore, this paper prepared EUTOL by selecting an epoxy resin dosage of 40%, adopting AC-5 gradation, and substituting steel slag for natural aggregate by an equal volume replacement method. Relevant experiments tested its pavement performance.

#### 3.3.1. Skid-Resistance Durability

**Figure 15:**
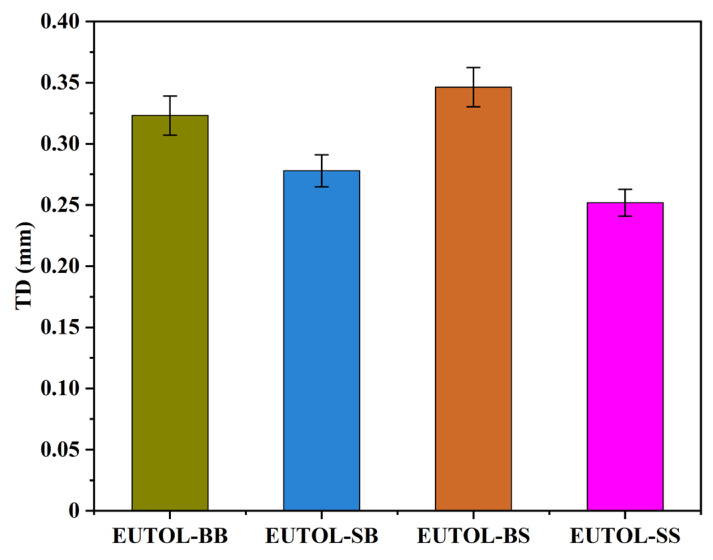
TD of different ETOULs.

**Figure 16:**
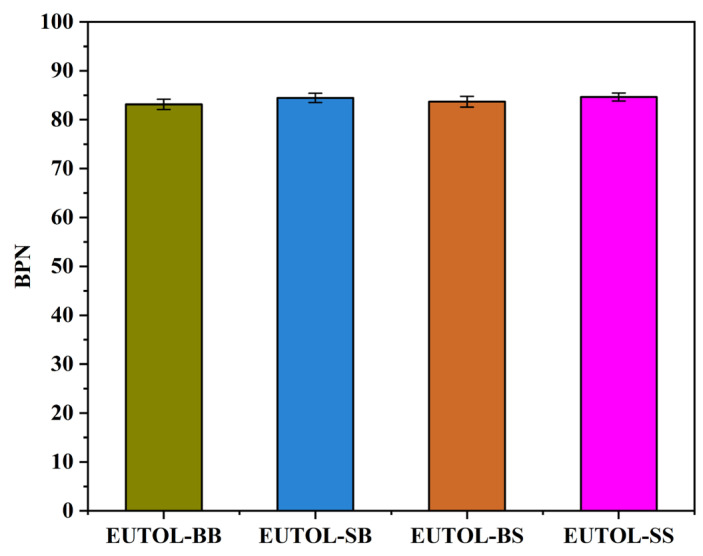
BPN of different ETOULs.

The Texture Depth Test (TD) and British Pendulum Number Test (BPN) of this pavement are two indicators of the skid resistance of asphalt pavements, which are related to the safety of driving and have an essential influence on the use of the pavement and are related to the roughness, skid resistance, and drainage performance of the pavement. Therefore, TD and BPN are chosen to characterize the skid-resistance durability of EUTOL in this paper. The test results are shown in Figure 15 and Figure 16. The TD of the four EUTOLs, from largest to smallest, is EUTOL-BS > EUTOL-BB > EUTOL-SB > EUTOL-SS. The results show that replacing basalt fine aggregate with steel slag fine aggregate decreases the TD of UTOL. This is mainly due to the higher proportion of powder particles smaller than 0.15 mm in steel slag fine aggregate compared to basalt fine aggregate. Although the effect of aggregate substitution can be reduced by using volumetric substitution, the fine aggregate is made up of multiple grades of aggregates, which will cause a slight effect. Despite this, the differences between the four EUTOLs are only within 0.1 mm. As depicted in Figure 16, the BPN of the four EUTOLs follows a descending order: EUTOL-SS > EUTOL-SB > EUTOL-BS > EUTOL-BB. This is attributed to steel slag aggregates’ better angularity and abrasion resistance than basalt aggregates. The rough surface texture of steel slag aggregate also contributes to increased roughness and distinct surface abrasion, further enhancing the BPN.

#### 3.3.2. High-Temperature Performance

**Figure 17:**
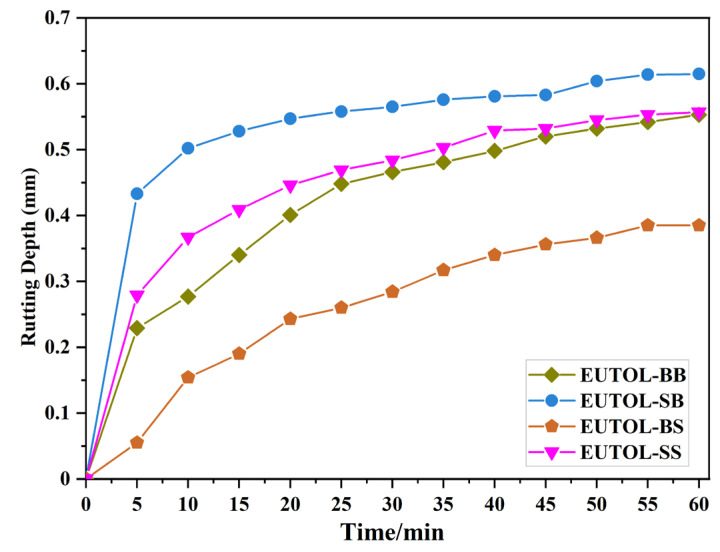
Rut depth versus time curves of different ETOULs.

This study analyzes EUTOL’s high temperature performance using a high temperature rutting test at an experimental temperature of 60 °C. In order to better match the road surface condition, a layered structure was used in this experiment, with 3 cm of AC-13 basalt selected for the lower layer and 2 cm of EUTOL selected for the upper surface. The test results are shown in Figure 17. EUTOL deformations at 60 min were in descending order of EUTOL-BS > EUTOL-BB > EUTOL-SS > EUTOL-SB. It showed that using steel slag fine aggregate increased EUTOL rutting depth, while using steel slag coarse aggregate decreased EUTOL rutting depth.

**Figure 18:**
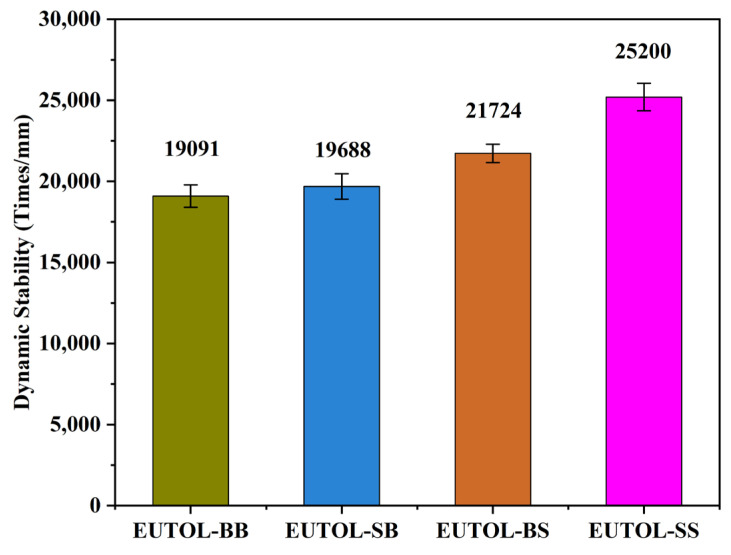
ETOUL’s rutting test results.

This is because the roundness of steel slag fine aggregate is larger than that of natural aggregate, and the fine-grained steel slag will form a more compact skeleton structure in one step and undergo greater deformation under external extrusion. In contrast, the steel slag coarse aggregate particle shape is irregular and multi-angled, with high internal friction between aggregates, making forming a mutually embedded skeleton support structure easy. The dynamic stability of the four EUTOLs is EUTOL-SS > EUTOL-BS > EUTOL-SB > EUTOL-BB in descending order, as shown in Figure 18. EUTOL’s high-temperature characteristics can be enhanced by substituting steel slag for basalt. Steel slag is complex, has a porous surface, and is alkaline, so it binds well to EA. And it is hard. However, the smallest EUTOL-BB also achieved dynamic stability of 19,091, indicating that EUTOL prepared using EA has good high-temperature performance.

#### 3.3.3. Low-Temperature Performance

**Figure 19:**
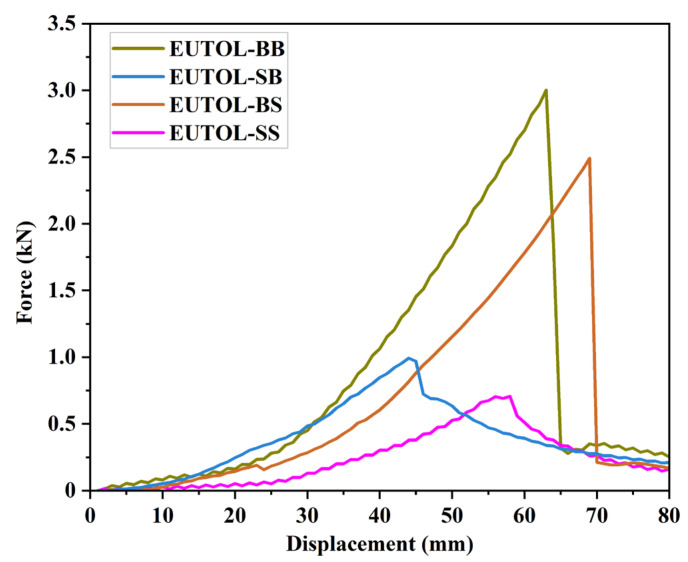
Relationship curve between displacement and load of different ETOULs.

This study tested the EUTOL’s low-temperature fracture resistance by the low-temperature trabecular test, shown in Figure 19 and Table 6. The damage load of EUTOL-BB was the largest at 3 N. In contrast, EUTOL-SB, EUTOL-BS, and EUTOL-SS damage loads were 33.1%, 83%, and 23.6% of EUTOL-BB, respectively. It indicated that basalt aggregate substitution by steel slag reduces EUTOL’s low-temperature strength; fine-aggregate steel slag also damages EUTOL’s low-temperature flexural properties more significantly than coarse steel slag. For different EUTOLs, the maximum bending strain and flexural tensile strength showed various tendencies, as shown in Table 6. The maximum bending strain is maximum for EUTOL-BS mixes and minimum for EUTOL-SB mixes. This phenomenon indicates that adding steel slag coarse aggregate improves EUTOL’s low-temperature deformation resistance. However, using fine-aggregate steel slag leads to a decline in EUTOL’s maximum bending tensile strain. The EUTOL’s flexural stiffness modulus (ratio of flexural strength to maximum flexural strain) is, in descending order, EUTOL-BB > EUTOL-BS > EUTOL-SB > EUTOL-SS. The flexural modulus is lowered when steel slag is used instead of basalt. The results show that the use of steel slag fine aggregate significantly reduces the flexural modulus.

#### 3.3.4. Water Stability Performance

**Figure 20:**
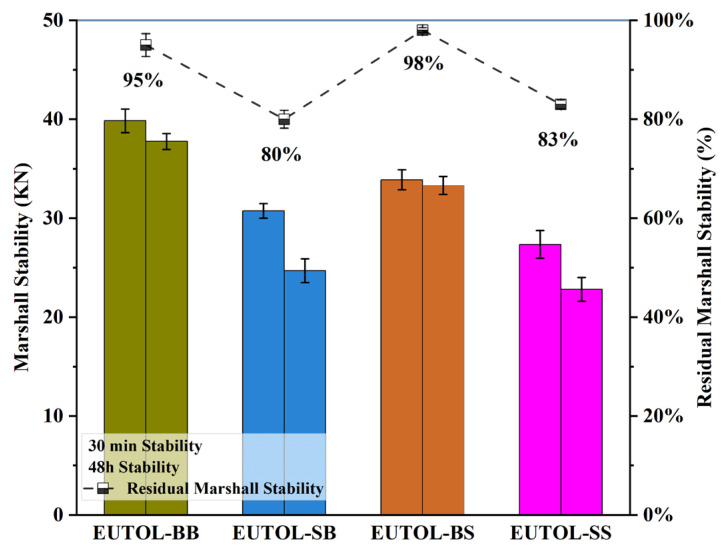
The Marshall strength and residual stability ratio of different ETOULs.

**Figure 21:**
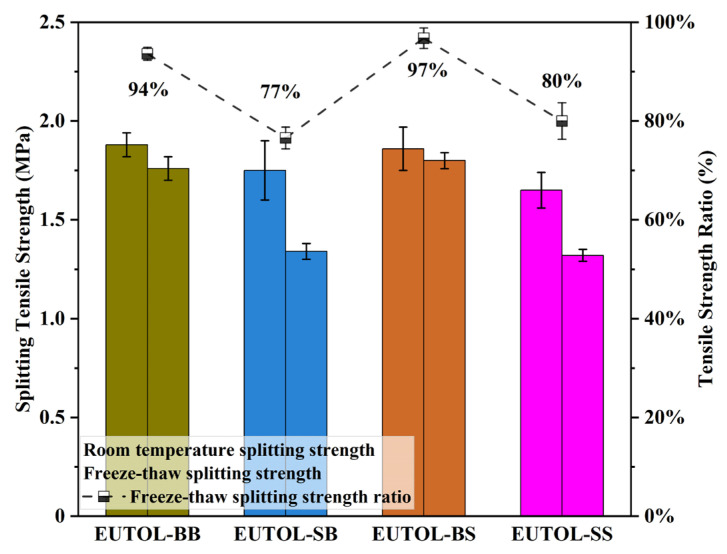
The splitting strength and freeze–thaw splitting ratio of different ETOULs.

In this study, the water damage resistance of EUTOL was tested using the water immersion Marshall test and freeze–thaw splitting test. The outcomes of the immersion Marshall test are depicted in Figure 20, while the results obtained from the freeze–thaw splitting test are illustrated in Figure 21. The results of both the residual Marshall stability ratings (MS) and the freeze–thaw split tensile strength ratings (TSR) are EUTOL-BS > EUTOL-BB > EUTOL-SS > EUTOL-SB. The data show that using coarse steel slag aggregate enhances EUTOL’s water damage resistance, but using fine steel slag aggregate decreases EUTOL’s water damage resistance. Moreover, the weakening effect of steel slag fine aggregate is more significant. Steel slag coarse aggregate has better adhesion with EA because it has a porous surface and is a strong alkaline aggregate. In steel slag fine aggregate in long-term water immersion, f-CaO will expand in contact with water, and white calcium hydroxide is generated on the aggregate surface, reducing water stability. One of the causes is the high dust content in the steel slag fine aggregate. The TSR of the steel slag asphalt mixes was more significant than the decrease in MS due to the more harsh conditions of the freeze–thaw splitting test than the water immersion Marshall stability test. Steel slag asphalt mixes showed a more significant decrease in TSR than MS. The reason is that the TSR specimens had a lower striking number, a larger void ratio, and more harsh conditions in the test. There are important influences on the EUTOL performance under warm and wet loading.

#### 3.3.5. Fatigue Performance

**Figure 22:**
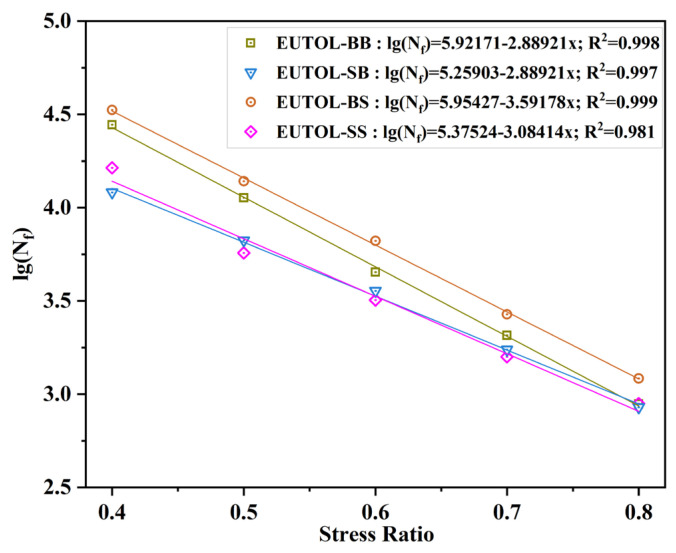
Relationship between fatigue life and stress level of different EUTOLs.

In this study, the fatigue durability performance of EUTOL was tested by selecting a stress ratio of 0.4–0.8 using the semicircular bending tensile test (SCB). The results are shown in Figure 22. The fatigue lifetimes of diffident EUTOL are as follows: EUTOL-BS > EUTOL-BB > EUTOL-SS > EUTOL-SB. At a stress ratio of 0.6, EUTOL-BS improved by 47%, EUTOL-SB decreased by 21%, and EUTOL-SS decreased by 29% compared to EUTOL-BB. The results indicate that the durability of EUTOL prepared using steel slag coarse aggregate instead of natural aggregate depends mainly on the choice of steel slag particle size. Using steel slag fine aggregate significantly reduces EUTOL fracture strength and fatigue life. In contrast, using steel slag coarse aggregate significantly improves the fatigue performance of the EUTOL. Steel slag coarse aggregate has a rough surface with internal frictional resistance and high hardness and is, therefore, more suitable for embedding and extrusion than basalt coarse aggregate, which helps to resist fatigue damage caused by repeated loading stress accumulation. On the other hand, steel slag fine aggregate has a more rounded surface and sharp edges than basalt, which is less effective for embedding and extrusion and is not conducive to resisting fatigue damage caused by repeated loading stress accumulation. Therefore, using steel slag coarse aggregate can increase EUTOL’s fatigue resistance.

## 4. Conclusions

In this paper, a UTOL with high toughness and durability is investigated. The micromorphology of EA with different epoxy resin dosages was observed using fluorescence analysis. The physical and rheological properties of EA were also investigated with different dosages. The curing behavior of the optimum dosage of EA also characterizes it. In addition, in this study, EUTOL was prepared using steel slag aggregate instead of natural aggregate, and the influences of different particle sizes of steel slag on the road performance and long-term durability of EUTOL were investigated. The main conclusions drawn from this study are as follows:

1. The results of fluorescence analysis experiments show that the particle size of epoxy resin particles gradually becomes larger when the EA dosage increases. However, when the EA content exceeds 40%, the epoxy resin particles undergo agglomeration behavior, decreasing the area share of the epoxy resin particles.
2. The doping of the epoxy resin significantly increased the EA’s softening point but decreased the EA’s needle penetration and ductility.

In the meantime, the elastic component of EA increases, and the high-temperature rheology is significantly improved, reflecting the excellent high-temperature rutting resistance.

The optimum doping amount of the epoxy resin was finally determined to be 40%.

3. DSC and FILT experiments showed that the EA cured at 94 °C to 114 °C. The EA was cured at 120 °C in three phases.

At 120 °C, the curing behavior of 40% EA is divided into three phases: 0–4 h for accelerated curing, with 69% of the reaction complete; 4–12 h for sustained growth, with 95% of the reaction complete; and after 12 h for slow curing.

4. The viscosity–time curve shows. At 150°, UTOL prepared with 40% EA has sufficient time for construction processes such as transportation, paving, and compaction.
5. Using steel slag coarse aggregate instead of natural coarse aggregate improves the slip resistance, high-temperature performance, low-temperature performance, water stability, and durability of EUTOL. At the same time, using steel slag fine aggregate reduced the performance of EUTOL.

Considering the macroscopic and microscopic properties of EA and the pavement performance of different grain sizes of steel slag in place of natural aggregates, it is recommended that 40% of EA and coarse steel slag aggregate be used to prepare EUTOL.

## Figures and Tables

**Table 1:** The performance indexes and test results of 70# base asphalt.

Test Indexes	Test Results	Technical Requirements	Test Methods [37]
Penetration (25 °C, 100 g, 5 s, 0.1 mm)	68.7	60~80	T0604-2011
Softening point (°C)	48.9	>46	T0606-2011
Ductility (15 °C, 5 cm/min, cm)	>150	≥100	T0605-2011
viscosity (135 °C)	207	≥180	T0607-2011
Specific gravity (25 °C)	1.039	-	T0603-2011

**Table 2:** The performance indexes and test results of epoxy resin.

Test Indexes	Component A Test Results	Technical Requirements	Component B Test Results	Technical Requirements	Test Methods
Viscosity (23 °C, Pa·s)	1.35	1~5	0.155	0.1~0.8	ASTM D445 [38]
Epoxide equivalent weight	187	190~220	55.2	180~220	ASTM D1652 [39]
Flashpoint (°C)	233	≥230	234	≥145	ASTM D92 [40]
Specific gravity (23 °C)	1.163	1.00~1.20	1.002	0.8~1.0	ASTM D1475 [41]
Asphalt light component (%)	-	-	67	-	-
Appearances	Light yellow transparent liquid	-	Dark brown liquid	-	visual assessment

**Table 3:** The performance indexes and test results of aggregate.

Test Indexes	Fine Aggregate		Coarse Aggregate		Technical Requirements	Test Methods [42]
	Steel Slag	Basalt	Steel Slag	Basalt		
Stone crushing value (%)	12.9	14.2	12.5	15.6	≤26	JTGE42 T0316
Los Angeles wear value (%)	15.6	14.1	11.8	13.3	≤28	JTGE42 T0317
Water absorption (%)	1.2	0.92	1.4	0.86	≤2.0	JTGE42 T0308
Apparent density	3.22	2.910	3.49	2.944	≥2.6	JTGE42 T0605

**Table 4:** Relevant test apparatuses and parameters of road performance of asphalt.

Test Name	Specimen	Test Temperature	Performance	Methods [37]	Photograph
Penetration	100 g, 5 s.	25 °C	Consistency performance	T 0604-2011	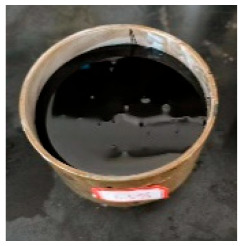
Softening Point	30 g	5 °C	Shifting performance	T 0606-2011	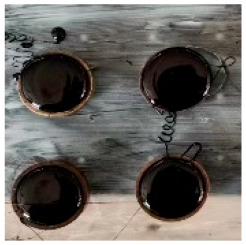
Ductility	5 g	15 °C	Plasticizing performance	T 0605-2011	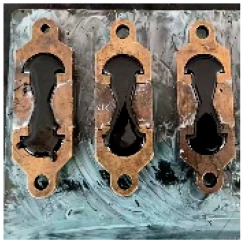
Viscosity	30 g	110–170 °C	Viscosity performance	T 0625-2011	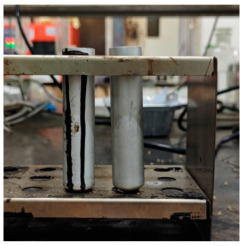
Dynamic Shear Rheometer	0.6 g	52–88 °C	rheological performance	T 0628-2011	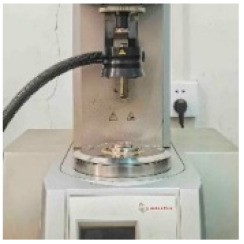

**Table 5:** Relevant test apparatuses and parameters of road performance of asphalt mixtures.

Test Name	Specimen Size	Test Temperature	Performance	Methods [37]	Photograph
Texture Depth Test (TD)	300 mm × 300 mm × 50 mm	25 °C	Skid-resistance durability	T0962-2019 [43]	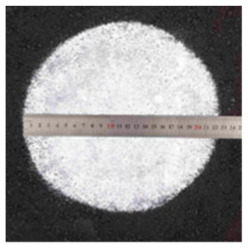
British Pendulum Number Test (BPN)	300 mm × 300 mm × 50 mm,	25 °C	Skid-resistance durability	T0964-2019 [43]	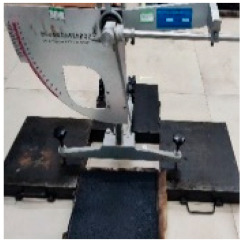
Wheel tracking Test	300 mm × 300 mm × 50 mm	60 °C	High- temperature	T 0719-2011	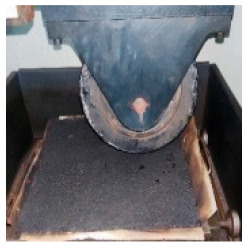
Freeze–thaw Split Test	Φ101.6 mm × 63.5 mm	25 °C	Water stability	T 0716-2011	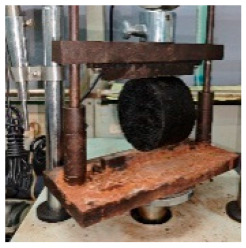
Immersion Marshall Test	Φ101.6 mm × 63.5 mm	25 °C	Water stability	T 0709-2011	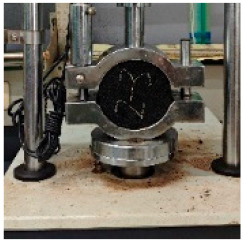
Three-point bending Test.	250 mm × 30 mm × 35 mm	−10 °C	Low temperature	T 0715-2011	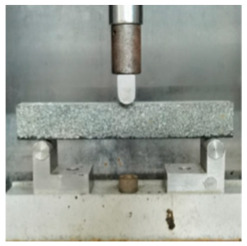
Indirect tensile fatigue test	Φ101 mm, 25 mm	15 °C	Fatigue resistance	AASHTO TP 105-2020	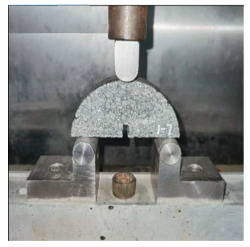

**Table 6:** Low-temperature trabecular results for different ETOULs.

The Type of Aggregate	Test Temperature (°C)	Peak Load (N)	Maximum Flexural Strain (με)	Flexural Stiffness Modulus (MPa)
EUTOL-BB	−10	3003	2798	7791
EUTOL-SB	−10	992	2440	4348
EUTOL-BS	−10	2492	3059	6161
EUTOL-SS	−10	707	2547	2606

## Data Availability

The data are available from the corresponding author upon reasonable request.

## References

[1] Li Q., Shen A., Guo Y., Wu J., Shi Y. (2024). Review of Evaluating Asphalt Pavement Structure Integrity and Strength with Rayleigh Wave Methods: Techniques, Applications, and Trends. Measurement.

[2] Pipintakos G., Sreeram A., Mirwald J., Bhasin A. (2024). Engineering Bitumen for Future Asphalt Pavements: A Review of Chemistry, Structure and Rheology. Mater. Des..

[3] Yang C., Wu S., Cui P., Amirkhanian S., Zhao Z., Wang F., Zhang L., Wei M., Zhou X., Xie J. (2022). Performance Characterization and Enhancement Mechanism of Recycled Asphalt Mixtures Involving High RAP Content and Steel Slag. J. Clean. Prod..

[4] Xu H., Zou Y., Airey G., Wang H., Zhang H., Wu S., Chen A. (2024). Wetting of Bio-Rejuvenator Nanodroplets on Bitumen: A Molecular Dynamics Investigation. J. Clean. Prod..

[5] World Steel Association (2024). Steel Statistical Yearbook 2023.

[6] Guo M., Zhang R., Du X., Liu P. (2024). A State-of-the-Art Review on the Functionality of Ultra-Thin Overlays Towards a Future Low Carbon Road Maintenance. Engineering.

[7] Abaza K.A., Ashur S.A. (1999). Optimum Decision Policy for Management of Pavement Maintenance and Rehabilitation. Transp. Res. Rec..

[8] Xie J., Chen J., Hu L., Wu S., Wang Z., Li M., Yang C. (2023). Preparation, Thermochromic Properties and Temperature Controlling Ability of Novel Pellets in Ultra-Thin Wearing Course. Constr. Build. Mater..

[9] Wang J., Sun J., Luo S., Li Q. (2022). Laboratory and Field Performance Evaluation of High-Workability Ultra-Thin Asphalt Overlays. Materials.

[10] Ding L., Wang X., Zhang K., Zhang M., Yang J., Chen Z. (2021). Durability Evaluation of Easy Compaction and High-Durability Ultra-Thin Overlay. Constr. Build. Mater..

[11] Fan Z., Wang C., Li Y., Feng L., Chen Q. (2024). State of the Art Review on Fog Seal for Asphalt Pavement: Material Composition, Classification, Performance Evaluation, and Mechanism Analysis. Prog. Org. Coat..

[12] Saghafi M., Tabatabaee N., Nazarian S. (2019). Performance Evaluation of Slurry Seals Containing Reclaimed Asphalt Pavement. Transp. Res. Rec..

[13] Zhan Y., Luo Z., Lin X., Nie Z., Deng Q., Qiu Y., Wang T. (2023). Pavement Preventive Maintenance Decision-Making for High Antiwear and Optimized Skid Resistance Performance. Constr. Build. Mater..

[14] Yu J., Feng Z., Chen Y., Yu H., Korolev E., Obukhova S., Zou G., Zhang Y. (2024). Investigation of Cracking Resistance of Cold Asphalt Mixture Designed for Ultra-Thin Asphalt Layer. Constr. Build. Mater..

[15] Hajj R., Filonzi A., Smit A., Bhasin A. (2019). Design and Performance of Mixes for Use as Ultrathin Overlay. J. Transp. Eng. Part B Pavements.

[16] Zheng X., Chen Y., Xu W., Zhang Z., Sun G., Wang T. (2023). Long-Term Performance Analysis of Epoxy Resin Ultra-Thin Wearing Course Overlay on Cement Concrete Pavement. Coatings.

[17] Abellán-García J., Carvajal-Muñoz J.S., Ramírez-Munévar C. (2024). Application of Ultra-High-Performance Concrete as Bridge Pavement Overlays: Literature Review and Case Studies. Constr. Build. Mater..

[18] Do M.-T., Tang Z., Kane M., De Larrard F. (2009). Evolution of Road-Surface Skid-Resistance and Texture Due to Polishing. Wear.

[19] Chen D.H., Scullion T. (2015). Very Thin Overlays in Texas. Constr. Build. Mater..

[20] Hu M., Li L., Peng F. (2019). Laboratory Investigation of OGFC-5 Porous Asphalt Ultra-Thin Wearing Course. Constr. Build. Mater..

[21] Cui W., Wu K., Cai X., Tang H., Huang W. (2020). Optimizing Gradation Design for Ultra-Thin Wearing Course Asphalt. Materials.

[22] Buddhavarapu P., Banerjee A., Prozzi J.A. (2013). Influence of Pavement Condition on Horizontal Curve Safety. Accid. Anal. Prev..

[23] Zhou D., Liang R., Kang Y. (2023). A Review of Chemo-Rheological and Thermo-Rheological Investigations on Epoxy Asphalt Cementitious Materials. Constr. Build. Mater..

[24] Zhang Z., Liang J., Hu J., Li J., Ni F. (2022). Characterizing the Curing Behavior and High-Temperature Performance of Epoxy-Resin Modified Asphalts. Constr. Build. Mater..

[25] Xiang Q., Xiao F. (2020). Applications of Epoxy Materials in Pavement Engineering. Constr. Build. Mater..

[26] Yu J., Cong P., Wu S. (2009). Laboratory Investigation of the Properties of Asphalt Modified with Epoxy resin. J. Appl. Polym. Sci..

[27] Zhang Z., Liu H., Ban X., Liu X., Guo Y., Sun J., Liu Y., Zhang S., Lei J. (2023). Thermosetting Resin Modified Asphalt: A Comprehensive Review. J. Traffic Transp. Eng. (Engl. Ed.).

[28] Huang W., Guo W., Wei Y. (2020). Prediction of Paving Performance for Epoxy Asphalt Mixture by Its Time- and Temperature-Dependent Properties. J. Mater. Civ. Eng..

[29] SprinkelI M.M. (1989). Performance of Multiple Layer Polymer Concrete Overlays on Bridge Decks. SP-116: Polymers in Concrete: Advances and Applications.

[30] Liu N., Liu L., Li M., Sun L. (2024). A Comprehensive Review of Warm-Mix Asphalt Mixtures: Mix Design, Construction Temperatures Determination, Performance and Life-Cycle Assessment. Road Mater. Pavement Des..

[31] Wang H., Qian J., Zhang H., Nan X., Chen G., Li X. (2024). Exploring Skid Resistance over Time: Steel Slag as a Pavement Aggregate—Comparative Study and Morphological Analysis. J. Clean. Prod..

[32] Cui P., Wu S., Xiao Y., Hu R., Yang T. (2021). Environmental Performance and Functional Analysis of Chip Seals with Recycled Basic Oxygen Furnace Slag as Aggregate. J. Hazard. Mater..

[33] Zhong T. (2024). Utilization of Steel Slag as Coarse Aggregate and Filler in Stone Mastic Asphalt (SMA) Mixture: Engineering Performance, Environmental Impact and Economic Benefits Analysis. J. Clean. Prod..

[34] Pattanaik M.L. (2022). Predicting the Abrasion Loss of Open-Graded Friction Course Mixes with EAF Steel Slag Aggregates Using Machine Learning Algorithms. Constr. Build. Mater..

[35] Zhang W. (2024). Testing and Evaluation for Skid Resistance of Steel Slag Asphalt Wearing Course Based on Surface Texture Characteristics. Constr. Build. Mater..

[36] Liapis I., Likoydis S. (2012). Use of Electric Arc Furnace Slag in Thin Skid–Resistant Surfacing. Procedia Soc. Behav. Sci..

[37] (2011). Standard Test Methods of Bitumen and Bituminous Mixtures for Highway Engineering, Engineering.

[38] (2021). Standard Test Method for Kinematic Viscosity of Transparent and Opaque Liquids (and Calculation of Dynamic Viscosity).

[39] (2019). Test Method for Epoxy Content of Epoxy Resins.

[40] (1992). Standard Test Method for Flash and Fire Points by Cleveland Open Cup.

[41] (2013). Standard Test Method for Density of Liquid Coatings, Inks, and Related Products.

[42] (2005). Test Methods of Aggregate for Highway Engineering.

[43] (2019). Field Test Methods of Highway Subgrade and Pavement.

[44] Liu X., Wu Z., Min Z., Zhang L. (2024). Investigation on the Preparation and Performances of Epoxy-Modified Asphalt Binder and Its Mixtures. Materials.

